# Dissecting the role of flagellar subunits in *C. difficile* mucosal colonization

**DOI:** 10.1128/jb.00428-25

**Published:** 2025-11-24

**Authors:** Baishakhi Biswas, Thi Van Thanh Do, Justin G. Perdomo, Jennifer M. Auchtung, Kurt H. Piepenbrink

**Affiliations:** 1 Department of Food Science and Technology, University of Nebraska-Lincoln 14719, Lincoln, Nebraska, USA; 2 Department of Biochemistry, University of Nebraska-Lincoln 315569, Lincoln, Nebraska, USA; 3 Nebraska Food for Health Center, University of Nebraska-Lincoln 822544, Lincoln, Nebraska, USA; 4 Center for Integrated Biomolecular Communication, University of Nebraska-Lincoln 602216, Lincoln, Nebraska, USA; 5 Department of Chemistry, University of Nebraska-Lincoln 207074, Lincoln, Nebraska, USA; University of Notre Dame, Notre Dame, Indiana, USA

**Keywords:** flagella, mucus, glycoprotein, host-pathogen interactions, *Clostridioides difficile*, cell culture, flagellar motility, phase variation

## Abstract

*Clostridioides difficile* is a common cause of acute gastrointestinal (GI) inflammation in mammals, which can have detrimental effects on host health. *C. difficile*-associated disease requires the secretion of high-molecular-weight toxins after colonization of the GI tract. The molecular mechanisms of GI colonization by *C. difficile* include potential interactions with host cells and the mucus layer formed from secreted mucin glycoproteins. *C. difficile* associates with the mucus layer *in vivo* and will associate with both epithelial cells and mucosal surfaces *in vitro*. Previously, we found a substantial defect in binding to mucosal surfaces for mutants of the major flagellar subunit, *fliC*, while mutation of the major subunit of type IV pili, *pilA1*, showed increased adhesion. To elucidate the mechanisms by which *C. difficile* interacts with *ex vivo* mucosal surfaces, we have measured swimming motility, mucosal adhesion, and levels of flagellation by transmission electron microscopy for mutants of flagellar and T4P genes in *C. difficile* R20291. We discovered that the *pilA1* mutant showed increased flagellation, while decreases in flagellation were found for *fliC, fliD,* and *flg*-Δ3 OFF (a phase-locked mutant with low transcription of the F3 flagellar operon), which were associated with both low swimming motility and low adhesion to mucosal surfaces. However, the reversed *flg*-Δ3 ON mutant showed increased flagellation without a significant increase in adhesion. We also found that the *fliC* mutant was defective in binding to mucus-secreting HT-29 MTX cells, and that decreased binding was not observed for other mutants with reduced flagellation. These results imply that at least two molecular pathways contribute to *C. difficile* mucosal adhesion. In addition to their direct roles encoding T4P and flagellar subunits, *pilA1* and *fliC* may contribute to regulatory networks governing other proteins relevant to mucosal adhesion.

**IMPORTANCE:**

In the context of previous work on *Clostridioides difficile* host adhesion by our groups and others, our results suggest that (i) at least two mechanisms exist for mucosal adhesion by *C. difficile*, potentially direct adhesion by flagella and another lectin-like adhesion regulated by flagellar components; (ii) mucosal binding can also contribute to *C. difficile* adhesion in 2D cell culture and could explain previous defects from *fliC* mutants; and (iii) levels of flagellation are largely insensitive to *fliC* transcription, implying that other factors limit flagellar production and that FliC levels may be regulated independently as part of regulatory networks within *C. difficile*.

## INTRODUCTION

*Clostridioides difficile*, a spore-forming, anaerobic, gram-positive bacterium linked to colitis and antibiotic-associated diarrhea, has garnered significant scientific interest due to its public health impact (1). According to the CDC, *Clostridioides difficile* infects around 224,000 hospitalized patients and causes nearly 13,000 deaths annually in the USA (2). Infections primarily occur in hospitals following antibiotic treatments that disrupt the gut microbiome (3). This disruption enables toxin-producing *C. difficile* to proliferate, resulting in *C. difficile* infection (4). As an obligate anaerobe, *C. difficile* vegetative cells cannot survive in aerobic environments outside a host (5). In response to environmental cues like nutrient limitation, quorum sensing, and other stress factors, *C. difficile* initiates sporulation to produce dormant spores capable of withstanding harsh conditions (5–7), on their own or as components of *C. difficile* biofilms (8).

For many organisms, adhesion to gastrointestinal (GI) cell surfaces is crucial for colonization and the production of virulence factors (9). Mucin proteins are heavily glycosylated and can be divided into those bound to the cell membrane and those that are secreted into extracellular space (although some membrane-bound mucins can be secreted through cleavage) (10, 11). Mucins, produced and secreted by goblet cells in the intestine, form a gel-like protective barrier that shields the intestinal epithelium from the gut microbiota (9, 11). In the colon, which is the primary site of *C. difficile* colonization (3), the mucus layer is often described as being divided into two layers, with an outer layer that serves as the site of bacterial colonization and an inner, sterile layer (12, 13). Mucin proteins have distinct roles, and those with known mechanisms from prior studies include MUC2, which supports the growth of commensal microbes, and MUC1, a membrane-bound mucin essential for defense against bacteria (12, 14, 15).

Flagella are crucial for bacterial swimming motility and promote gastrointestinal colonization in many bacterial species, acting as key virulence factors in early infection, as seen for GI pathogens *Escherichia coli*, *Salmonella Typhimurium*, *Helicobacter pylori,* and *Vibrio anguillarum* (16–19). For *C. difficile,* flagella have been shown to play essential roles in adhesion to and colonization of the gut (20). Prior studies have shown that *C. difficile* mutant strains lacking the major structural protein flagellin (FliC) and the flagellar cap (FliD) have reduced adhesion to mouse mucus (21) and exhibit a reduced capacity to adhere to the mouse cecum (21). However, naturally occurring aflagellate clade V *C. difficile* strains have been shown to adhere to mucosal surfaces *ex vivo* (22).

The mechanisms by which *C. difficile* interacts with the mucus layer and underlying epithelium to promote colonization are not well understood, even though this association is known to occur. Our group and others have previously demonstrated that *C. difficile* is able to bind directly to mucins from human colonic cell lines and porcine gastric and colonic tissues when tested using *ex vivo* mucosal surfaces (22, 23). We demonstrated that *fliC* mutant strains had reduced adherence to gastrointestinal mucins compared to the parent strain. This finding suggested that interactions between host mucins and *C. difficile* flagella facilitate the initial host attachment of *C. difficile* to host cells and the production of mucus. However, as an *fliC* mutation is known to have pleiotropic effects on gene expression in *C. difficile* (24)*,* we could not exclude an indirect mechanism by which FliC could mediate mucin binding. We also showed that a mutant unable to produce the type IV pilus due to a mutation in *pilA1*, the gene encoding the major structural protein of the type IV pilus, bound at higher levels than wild-type bacteria; although we could find no direct explanation for increased adherence from the loss of an adhesive appendage.

**Fig 1:**
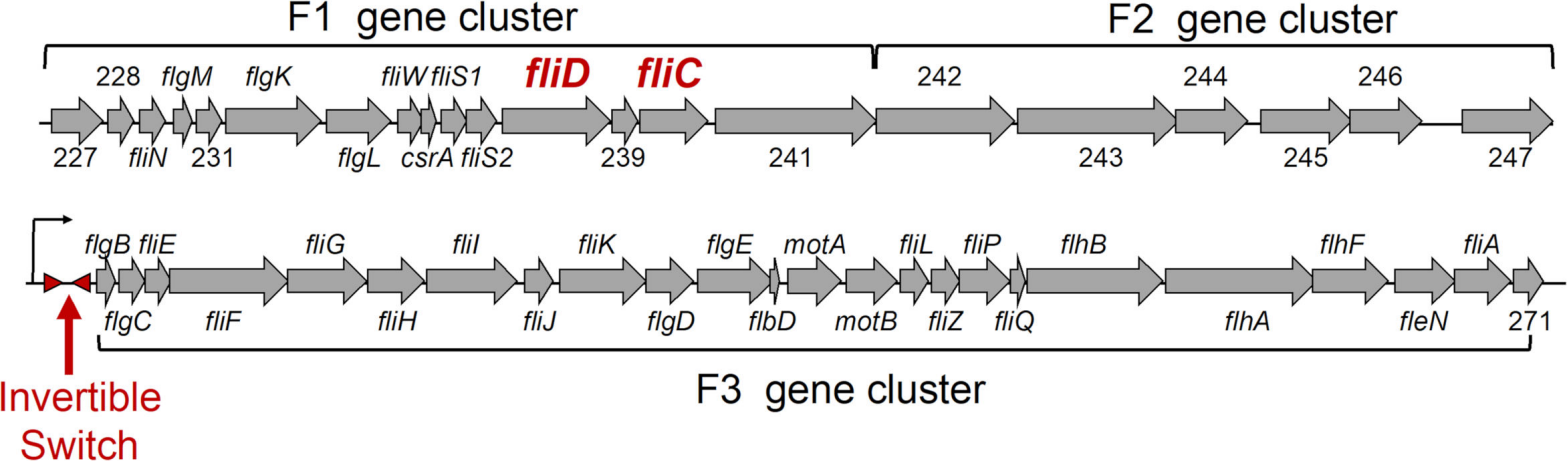
Flagellar operons in *C. difficile* strain R20291. Flagella production is governed by three major gene clusters in *C. difficile*, F1, F2, and F3. These gene clusters encode structural proteins, regulators, transporters, modification enzymes, and genes of unknown function (locus tags indicated by numbers). In *C. difficile* R20291, expression of the F3 gene cluster is regulated by an invertible regulatory switch that allows or inhibits gene expression. The presence of this invertible switch varies across strains of *C. difficile*.

Previous work from Trzilova et al. showed that motility and toxin production are regulated by phase variation in *C. difficile* strain R20291 (25, 26). They discovered an invertible flagellar switch upstream of the F3 gene cluster, which contributes to regulation of the F3 operon that is essential for flagella formation (Fig. 1). To further characterize the effects of phase variation on *C. difficile* physiology and pathogenesis, the Tamayo group created small deletions in the invertible switch region to generate *flg*-Δ3 ON and *flg*-Δ3 OFF mutants (25, 27). The *flg*-Δ3 ON mutant showed greater swimming motility and toxin production *in vitro* and caused greater weight loss and a higher level of colonization in an *in vivo* mouse model. However, in a hamster model of infection, greater toxin production by *flg*-Δ3 ON did not lead to higher levels of colonization or acute disease. These findings suggest that naturally occurring variants generated by flagellar phase switching also have distinct potentials for disease progression (25).

To probe the potential roles played by flagella in mucosal attachment, this study examined how mutations to multiple flagellar genes (*fliC*, *fliD*), *pilA1,* and phase-locked mutants *flg*-Δ3 ON and *flg*-Δ3 OFF in *C. difficile* R20291 impacted flagellation, macroscopic swimming motility, and adhesion to *ex vivo* mucosal surfaces. We found that, like the parent strain*, flg*-Δ3 ON *and pilA1* had peritrichous flagella, while *fliC*, *fliD*, and *flg*-Δ3 OFF were aflagellate; *pilA1* and *flg*-Δ3 ON were hyperflagellated compared to the parent strain. We observed that flagella were required for macroscopic swimming motility, but hyperflagellation did not increase motility. The *pilA1* mutant exhibited a significant increase in mucosal adhesion, whereas the *flg*-Δ3 ON strain exhibited a minor decrease in binding compared to the wild type, and all aflagellate strains showed significantly decreased binding. Similar roles for *fliC* in mucin adherence were seen in a cell culture-based model of mucin adherence. Measurements of transcription through qRT-PCR data indicate that hyperflagellation in *pilA1* or *flg*-Δ3 ON is not due to increased *fliC* expression and that *fliC* expression was significantly lower in both the hyperflagellated *flg*-Δ3 ON and the aflagellate *flg*-Δ3 OFF strain compared to the wild type (the *fliD* mutant had *fliC* transcription levels comparable to the parent strain). Taken together, these results suggest that flagellation directly promotes adhesion, but that hyperflagellation alone is insufficient to promote strong binding, suggesting a role for other adhesins. There is a significant gap in understanding *C. difficile* colonization of the gut, particularly regarding its interactions with host cells and mucosa. Our findings here help to interpret previous reports of adhesion abnormalities in flagellin and pilin mutants, emphasizing the crucial role of flagellin in adherence to mucosal hydrogels.

## MATERIALS AND METHODS

### Bacterial strains and growth conditions

Wild-type and mutant strains of *Clostridioides difficile* R20291, a well-studied ribotype 027 clinical isolate, were used in this study (28). Wild-type *C. difficile* R20291 was a gift from Glen Armstrong (The University of Calgary), and the *pilA1* and *fliC* mutant strains were created by his group as previously described (22, 29). The R20291 *fliD* gene-interruption mutant was also created by Glen Armstrong using the CLOSTRON mutagenesis system, as described for the CD630 *fliD* mutant in Dingle et al. (30). Phase-locked mutants *flg*-Δ3 ON and *flg*-Δ3 OFF (15) were a generous gift from Rita Tamayo (University of North Carolina at Chapel Hill), as was her wild type R20291 strain. Brain Heart Infusion Supplemented (BHIS) medium was prepared from Difco BHI medium supplemented with 5 g/L yeast extract and was used for all growth assays. BHIS medium was prepared without cysteine supplementation to limit the potential for hydrogen sulfide production. For routine culture, BHIS agar plates were prepared with 1.5% wt/vol agar. All growth was performed at 37°C in an anaerobic chamber (Coy Laboratory Products) with an atmosphere of 90% nitrogen, 5% carbon dioxide, and 5% hydrogen. At the start of each experiment, *C. difficile* strains were inoculated from a glycerol stock onto pre-reduced BHIS agar plates. Details for subsequent growth steps unique to each assay are noted below.

### Preparation of artificial mucosal surfaces

Extraction and purification of porcine colonic mucin (from pig intestinal tracts generously provided by UNL’s Animal Science Department) were performed as previously described (22). Briefly, porcine colonic mucin, resuspended at a final concentration of 1 mg/mL in mucin coupling buffer (0.0308 M K_2_HPO_4_, 0.0192 M KH_2_PO_4_, 0.15 M NaCl, and 0.01 M EDTA), was used to coat glass coverslips (25 × 25 mm) using our previously published APTES-mediated coupling approach (22) with the following modifications. After modification with APTES and glutaraldehyde, each 25 × 25 mm glass coverslip was submerged in 3.5 mL of mucin solution in a 55 mm diameter polypropylene Petri dish (Eisco labs) and incubated overnight on an orbital shaker at 4°C. To ensure even mucin adsorption, coverslips were turned over, returned to the mucin solution, and placed back on the shaker the following day at 4°C for an additional 4 h. Next, coverslips were washed with phosphate-buffered saline (PBS; 137 mM NaCl, 2.7 mM KCl, 10 mM Na_2_HPO_4_, 1.8 mM KH_2_PO_4_, pH 7.4) and exposed to UV light for sterilization. Mucin-coated coverslips were stored at 4°C in a coverslip holder (Sigma-Aldrich) within a sterile container for future use (1).

### Bacterial adherence to mucosal surfaces

For all strains, 5 to 10 colonies were inoculated into BHIS broth and grown anaerobically at 37°C until mid-exponential phase (optical density at 600 nm = 0.5, approximately 4–6 h). Bacterial cells were centrifuged at 5,000 × *g* for 5 min, decanted under anaerobic conditions, then resuspended in anaerobic PBS at an OD_600_ = 0.5. An aliquot of the cell suspension was removed for determination of colony-forming units (CFU) through serial dilution and plating. Six milliliters of cell suspensions were added to each well of sterile, six-well cell culture plates (Costar, Corning Incorporated) that contained mucin-coated glass coverslips that had been pre-reduced for 2 h. Cells were incubated with coverslips for 1 h at 37°C. Binding was also assessed to control glass coverslips that were treated as described above, save that the incubation was in mucin coupling buffer without added mucin. The coverslips were carefully rinsed four times with PBS to eliminate any non-adherent bacteria. Rinsing was performed by sequential dipping of coverslips into sterile, square polypropylene containers (4 cm^2^) that contained 40 mL of PBS. To measure the adherent bacterial population, coverslips were then immersed in a 0.25% trypsin-EDTA solution (Gibco) at 37°C for 10 min. Trypsin activity was neutralized by adding two volumes of BHIS broth, and levels of adherent cells were determined by serial dilution and plating of cells onto BHIS agar plates, which were incubated at 37°C for 24 h to facilitate colony growth and enumeration. After 24 h, the number of colonies per plate was counted, and percent binding was calculated as the CFU per milliliter of adherent bacteria divided by the CFU per milliliter inoculum of bacteria.

### Bacterial adherence to HT-29 and HT-29 MTX 2D cell culture

Authenticated HT-29 and HT-29 MTX-E12 cell lines were obtained from Sigma-Aldrich and cultured in 75 cm² plastic flasks (Fisher Scientific). HT-29 cells were maintained in Roswell Park Memorial Institute 1640 (RPMI 1640) medium with L-glutamine and sodium bicarbonate (Sigma-Aldrich), supplemented with 10% (vol/vol) fetal bovine serum (FBS), 1% penicillin-streptomycin solution (10,000 U/mL, Gibco), and 1% amphotericin B (250 µg/mL, Gibco). HT-29 MTX-E12 cells were cultured in Dulbecco’s Modified Eagle Medium (4.5 g/L glucose and L-glutamine, without sodium pyruvate), supplemented with 10% (vol/vol) FBS, 1% penicillin-streptomycin solution (10,000 U/mL, Gibco), 1% amphotericin B (250 µg/mL, Gibco), 1% MEM non-essential amino acids (100×, Gibco), and 1% GlutaMAX (100×, Gibco).

For use in the experiments, 12-well tissue culture plates (Fisher Scientific) were seeded with 100,000 cells per well. HT-29 and HT-29 MTX-E12 cells were used at passages 8–13 and 57–59, respectively. The culture medium was replaced 3 days after seeding and subsequently every 2 days for a total of 18 days. All cultures were maintained at 37°C in a humidified atmosphere of 5% CO_2_ and 95% air. A 12-well plate containing confluent monolayers of HT-29 and HT-29 MTX-E12 cells was gently washed twice with pre-warmed, bicarbonate-free, phenol red-free Hanks’ Balanced Salt Solution (HBSS). For both strains of *C. difficile* R20291 (wild-type and *fliC* mutant), 5 to 10 colonies from BHIS agar plates (grown anaerobically at 37°C for 24 h) were inoculated into BHIS broth and cultured overnight under anaerobic conditions at 37°C. The overnight cultures were centrifuged at 4,700 × *g* for 8 min at 20°C, and the resulting bacterial pellets were resuspended in anaerobic HBSS in the original culture volume. A 1 mL aliquot of this bacterial suspension was anaerobically added to each well of the 12-well plate containing the washed monolayers. The plates were incubated anaerobically at 37°C for 2 h. Following incubation, supernatants were collected, serially diluted, and plated on BHIS agar to enumerate viable bacteria after the 2 h incubation. To quantify bound bacteria, the remaining supernatant was discarded, and the wells were washed three times with HBSS to remove unbound bacteria. The monolayers were then scraped into 1 mL of HBSS, thoroughly mixed, and serially diluted before plating onto BHIS agar plates. After 24 h of anaerobic incubation at 37°C, CFU were counted. The percentage of adherence was calculated as the CFU per milliliter of bound bacteria divided by the total CFU per milliliter of bound and viable bacteria after 2 h of incubation, multiplied by 100. To determine bacterial attachment to plastic wells, the same procedure was conducted using 12-well plates without cell monolayers. To assess the impact of flagellar motility on the adherence of R20291 strains (wild-type and *fliC* mutant), the infected plate was sealed with tape and removed from the anaerobic chamber. It was then centrifuged at 1,000 × *g* for 10 min at 25°C to sediment the bacteria onto the tissue culture cells. After centrifugation, the plate was returned to the anaerobic chamber and incubated at 37°C for 2 h. Subsequent steps were carried out following the same protocol described above.

### Reduction of extracellular mucus on HT-29 MTX-E12 cultures by pretreatment with N-acetylcysteine (NAC)

To investigate the contribution of extracellular mucus to the enhanced adherence of *C. difficile* R20291 strains, HT-29 and HT-29 MTX-E12 cell monolayers cultured for 18 days were pretreated with NAC, a mucolytic agent known to reduce adherent mucus layers in these cell lines (31, 32). A 20 mM NAC solution (Sigma-Aldrich) was prepared in PBS and adjusted to pH 7.5 using NaOH, as NAC exhibits optimal mucolytic activity within the pH range of 7.0–9.0 (33). The treated culture plates were returned to a cell incubator and agitated using an Infinity rocker (Next Advance) at 50 cycles per minute for 1 h, followed by two washes with PBS. For infection, the monolayers were further washed twice with anaerobic HBSS and subsequently infected with *C. difficile* R20291 strains to assess adherence as discussed above.

### Staining of cellular-associated mucins

To visualize mucins on the surface of HT-29 and HT-29 MTX-E12 cells, HT-29 and HT-29 MTX monolayers were cultured for 18 days, untreated and treated with NAC, in 12-well plates, washed three times with PBS, and fixed in freshly prepared Methacarn solution (60% methanol, 30% chloroform, 10% acetic acid) for 15 min at room temperature to preserve mucus integrity (34, 35). For acidic mucins, monolayers were treated with 3% acetic acid for 3 min and then stained with 1% Alcian Blue 8GX (Sigma-Aldrich) for 15 min with gentle agitation on an orbital shaker (VWR 1000) to prevent dye pooling. Excess dye was removed by washing with 3% acetic acid until clear, followed by three rinses with distilled water. Monolayers were then dehydrated sequentially with 70% ethanol, 96% ethanol, 100% ethanol, and 100% isopropanol. For neutral mucins, fixed cells were rinsed with distilled water and oxidized with 0.5% periodic acid (Electron Microscopy Sciences) for 5 min. After three rinses in distilled water, samples were incubated with Schiff’s reagent (Electron Microscopy Sciences) for 15 min and washed with tap water for 5 min. For a subset of cells stained with periodic acid-Schiff reagent, counterstaining was performed with Mayer’s hematoxylin (Electron Microscopy Sciences) for 1 min, followed by an additional 5 min wash in tap water and rinsing in distilled water until clear. Dehydration was carried out as described above for Alcian Blue staining. To preserve stained monolayers, 1 mL of 50% glycerol was added per well. Imaging was performed using the Invitrogen EVOS XL Core Imaging System.

### Macroscopic swimming motility assay

For swimming motility assays, BHIS agar plates were prepared with 0.3% (wt/vol) agar. Specifically, 100 mm diameter disposable plastic Petri plates (50 mm depth) were filled with 35 mL agar, allowed to solidify, and moved to the anaerobic chamber to pre-reduce for at least 24 h. At all times, 0.3% agar plates were stored in a covered container to limit evaporation. *C. difficile* strains were grown anaerobically on 1.5% BHIS agar plates for 48 h. For each strain, three to five single colonies were collected from the 1.5% BHIS agar plate with a sterile, disposable 1 µL inoculating loop and stabbed into the center of the pre-reduced 0.3% BHIS agar plates. Each strain was tested in triplicate in two independent experiments, with the exception of *fliC,* which was tested in duplicate in the second experiment. Following inoculation, plates were incubated anaerobically at 37°C, and the length and width of cell migration were measured every 24 h for 4 (experiment 1) or 5 (experiment 2) days. Visual inspection of the agar confirmed that cell migration occurred in the agar, rather than on top of the agar or below the agar on the surface of the plastic Petri plate. The extent of bacterial macroscopic swimming was reported as the area of migration (π × (length (mm)/2) × (width (mm)/2)).

### Measurement of flagellation by transmission electron microscopy (TEM)

**Fig 2:**
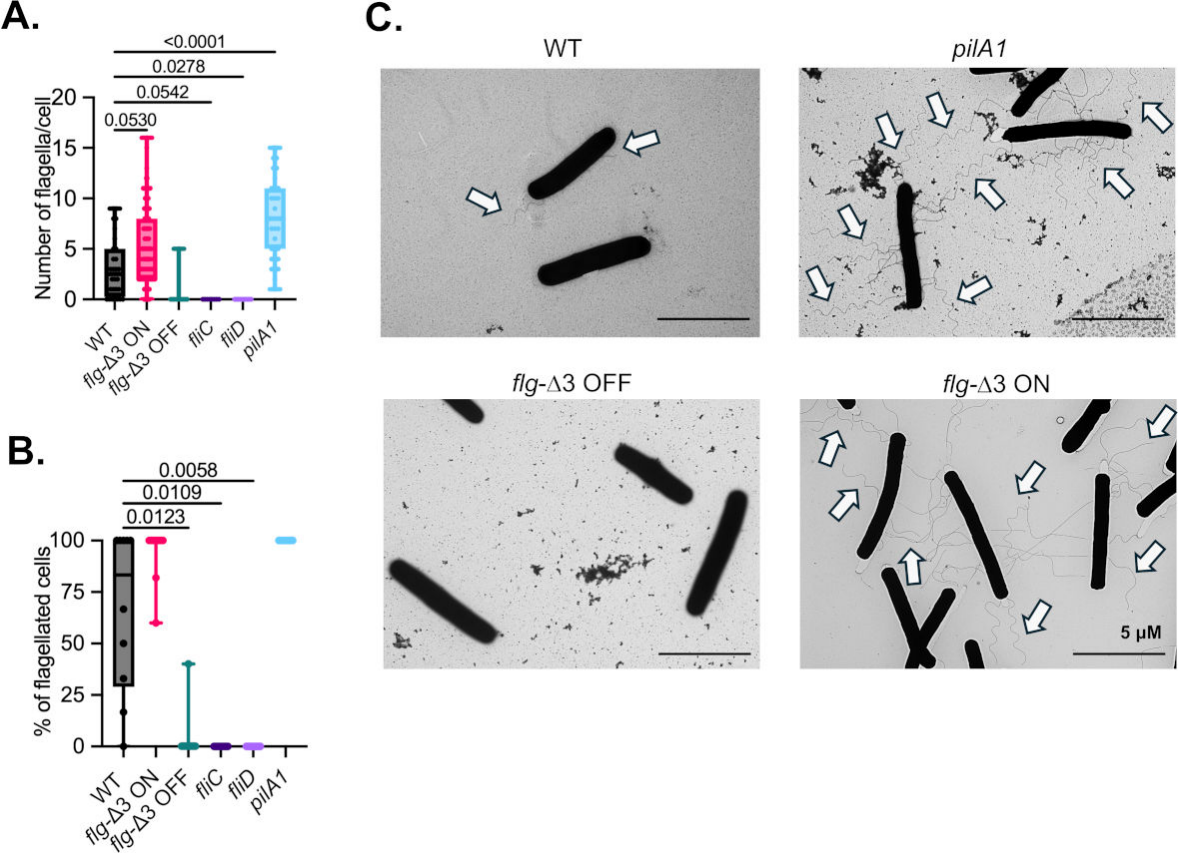
*flg-*Δ3 ON and *pilA1* mutations increase the number of flagella per cell. (**A**) The number of flagella per cell and (**B**) percentage of flagellated cells were determined from enumeration of TEM images. (**C**) Representative transmission electron microscopy images of wild-type, *pilA1, flg*-Δ3 ON*,* and *flg*-Δ3 OFF strains. Significance of differences from wild type was determined with Kruskal-Wallis testing with Dunn’s correction for multiple comparisons. All *P*-values less than 0.1 are shown.

The number of flagella on wild-type and mutant *Clostridioides difficile* R20291 cells was quantified using TEM. As described above, *C. difficile* strains were grown anaerobically in BHIS broth at 37°C for 4–6 h until OD_600_ = 0.5. Bacterial cultures were fixed by combining with an equal volume of 2.5% glutaraldehyde in 100 mM cacodylate buffer, pH 7.4, and incubating at room temperature for 1 h. Cells were washed twice in the same buffer. Samples were stored at 4°C until they were prepared for TEM examination at the UNL Center for Biotechnology’s Microscopy Research Core Facility (36). To set bacteria on TEM grids, a 30 µL droplet of bacterial suspension was placed on Parafilm and transferred to a 200-mesh carbon-formvar-coated copper grid by placing the grid, coated side down, in contact with the samples for 30–60 s. After that, excess bacterial suspension was wicked away by touching a piece of filter paper to the edge of the grid surface, and the samples were air-dried for 30 s. The grid was then placed with sample side down onto a droplet of stain solution (1% uranyl acetate) for 30–60 s. The excess staining solution was wicked from the grid following the staining. The grids with samples were air-dried for 10 min before they were examined at 80 kV under a Hitachi HT7800 TEM. Ten to 15 TEM micrographs were obtained per strain at multiple magnifications with 7,000× images used for quantification in Fig. 2.

### *fliC* expression measurement through qRT-PCR

**Fig 3:**
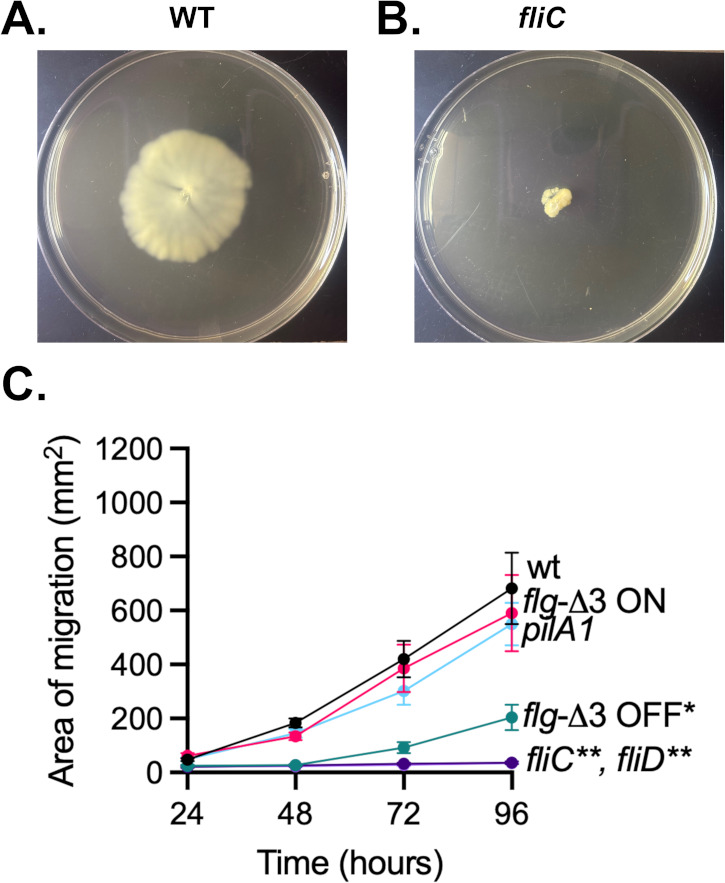
Differences in macroscopic swimming motility between R20291 strains observed over time. Representative images of (**A**) wild-type and (**B**) *fliC* mutant strains migrating through 0.3% BHIS agar are shown at 96 h. (**C**) Area of cell migration for each strain in 0.3% BHIS agar was assessed in triplicate over two independent experiments at the time points indicated, with mean and standard error of the mean plotted for each time point. Significance of differences from wild type of the area under the curve was determined by Kruskal-Wallis testing with Dunn’s correction for multiple comparisons, with *P*-values less than 0.05 reported. *, *P* < 0.05; **, *P* < 0.01.

Bacteria were grown from frozen stocks on BHIS agar plates for 24 h. Ten colonies were added to 14 mL of BHIS broth and incubated at 37°C until a cell growth of OD_600_ ~0.5 was reached, which occurred after 5–6 h of growth. Cells were then mixed with an equal volume of ice-cold methanol and centrifuged for 10 min at 3,000 × *g* at 4°C to pellet cells. Pellets were washed in 500 µL of Tris-EDTA (10 mM Tris-HCl, 1 mM EDTA, pH 7.5), centrifuged for 3 min at 4,000 × *g* at 4°C, and resuspended in 1 mL Buffer RLT obtained from the Qiagen RNeasy kit. A total of 0.1 mm silica-glass beads was added to samples, and cells were disrupted by bead beating for 1 min. The supernatant was carefully removed, and 1 vol of 70% ethanol was added to the lysate and mixed well by pipetting. RNA extraction then proceeded according to the Qiagen protocol with optional DNase I digestion (Qiagen). RNA concentration was determined by UV spectrometry, and quality was assessed by stable RNA (16S, 23S rRNA) band integrity following gel electrophoresis. qRT-PCR was performed in two steps. In the initial reverse transcription step, 5 µg of total RNA was incubated with 1 µL of 50 mM random hexamers (Invitrogen) and 1 µL of 10 mM dNTP mix (Invitrogen) in a total volume of 12 µL at 65°C for 5 min. Reverse transcription was then performed with Superscript II Reverse Transcriptase (Invitrogen). Specifically, 4 µL of 5× First-Strand Buffer, 2 µL of 0.1 mM DTT, and 1 µL of RNaseOUT Recombinant Ribonuclease Inhibitor (Invitrogen) were added to each reaction, which was then incubated at 25°C for 2 min. 1 µL of Superscript II reverse transcriptase was then added to each reaction and reactions were incubated at 25°C for 10 min, 42°C for 50 min, and 70°C for 15 min. One microliter of Ribonuclease H (Invitrogen) was then added to each tube to degrade RNA/DNA hybrids before initiating qPCR. qPCR was performed using primers designed to amplify a section of the *fliC* gene (*fliC* forward: GCA CAA AGT AAG TCT ATG G; *fliC* reverse: CAG ATA TAC CAT CTT GAA CG) as well as *rpoA,* which encodes the alpha subunit of RNA polymerase (*rpoA* forward: CAT GCT CTA TCA CAG GTG CAG; *rpoA* reverse: CA ACT CTT GTG TTT TCC ACA). Duplicate qPCR reactions were set up for each reverse-transcribed product. Twenty microliter reactions contained 1 µL of reverse-transcribed DNA, 300 nM forward and reverse primers, and 1× Power SYBR Green PCR mix (Thermo Fisher Scientific). Amplification was performed in a QuantStudio 3 PCR machine (Thermo Fisher Scientific) with an initial denaturation step for 10 min at 95°C, followed by 40 cycles of a 15 s denaturation step at 95°C, followed by a 60 s annealing and elongation step at 60°C. A previous version of this manuscript (https://www.biorxiv.org/content/10.1101/2025.01.02.631138v1) contained qRT-PCR data for *C. difficile* CD1015 mislabeled as *C. difficile* R20291 *flg*-Δ3 ON. When this error was discovered, all strain identities were verified by sequencing, and the data were all collected from sequence-verified strains. Control reactions lacking reverse transcriptase were performed to ensure that signals measured were from mRNA rather than DNA. All reactions lacking reverse transcriptase had C_t_ values more than 10 cycles higher than reactions with reverse transcriptase. Relative levels of *fliC* were determined through the ΔΔC_t_ method with the average of wild-type replicates used as a baseline for expression (Fig. 3).

### Analysis of data and evaluation of statistical significance

Data were visualized and the statistical significance was determined with GraphPad Prism version (10.0.2) (Cambridge, MA). Tests performed in each experiment are indicated in the figure legends. Significance values less than *P* < 0.05 are reported.

## RESULTS

### Disruption of *pilA1* impacts flagellation

The flagellum of *C. difficile* is a highly studied component that contributes to colonization (30). In a previous study, we demonstrated that the major structural protein of flagella, FliC, contributed to mucin adhesion. While *fliC* mutant cells exhibited reduced adherence to mucin hydrogels compared to wild-type cells, *pilA1* mutants, which lack the major structural protein of type IV pili, exhibited increased binding to mucosal surfaces. We hypothesized that this increased mucosal adhesion may be due to increased flagellation in a *pilA1* mutant. To test this hypothesis, TEM images were collected from wild-type and *pilA1 R*20291 strains, and the number of flagella per cell (Fig. 2A) and the proportion of flagellated cells (Fig. 2B) were measured. Previous studies of *C. difficile* R20291 identified two primary lineages that display significant phenotypic variations (28). Germane to this study, CRG0825 strains show peritrichous flagellation, while CRG2021 strains show a single polar flagellum. Our wild-type strain, which previous sequence analysis demonstrated was in the CRG0825 lineage, was peritrichously flagellated, with a mean number of flagella per cell of 3.1 ± 3.0. However, not all cells (67 ± 39%) were flagellated. No flagella were observed on cells from any strain in equivalent experiments using bacteria grown on solid media (Fig. S1). As we expected, the *fliC* mutant strain lacked flagella, as did cells with mutations in *fliD,* which encodes the flagellar cap. The percentage of flagellated cells and the mean number of flagella per cell both rose in the *pilA1* mutant to 100% and 8.1 ± 3.9, respectively, consistent with our hypothesis that the *pilA1* mutant is more highly flagellated.

We also assessed the number of flagella per cell and percent flagellation in cells where phase variation of the F3 operon was locked in the “ON” or “OFF” position due to a three-nucleotide deletion in the invertible switch (25). Consistent with prior observations (25)*, flg*-Δ3 OFF mutants were primarily aflagellate. In contrast, *flg*-Δ3 ON mutants were peritrichously flagellated, with a mean number of flagella per cell of 5.2 ± 3.7 (Fig. 2A) and a percentage of flagellated cells of 96 ± 11% (Fig. 2B), significantly higher than wild-type cells. Based on these results, we conclude that both *pilA1* and *flg*-Δ3 ON mutants are hyperflagellated, whereas *fliC, fliD, and flg*-Δ3 OFF mutants are primarily aflagellate.

### Macroscopic swimming motility is unaffected by hyperflagellation

To investigate the relationship between hyper- and hypoflagellation and swimming motility, we measured macroscopic swimming motility for the strains described above. Swimming motility was assessed by measuring the migration of cells through 0.3% BHIS agar over a 96 h incubation (Fig. 3). We observed that the mean distance moved for wild-type cells increased over time, with a final area of migration at 96 h of 682 ± 325 mm^2^. *flg*-Δ3 ON and *pilA1* mutants exhibited comparable final areas of migration of 591 ± 346 mm^2^ and 550 ± 192 mm^2^, respectively. Although hyperflagellation did not enhance macroscopic swimming motility, the *fliC* and *fliD* mutants were non-motile with areas of migration at 96 h of 35.6 ± 11.6 mm^2^ and 35.5 ± 5.05 mm^2^. For the first 48 h, motility was not significantly different between *flg*-Δ3 OFF and *fliC* mutant strains (*P* = 0.96, Mann-Whitney test), with areas of motility of 27.3 ± 7.17 mm^2^ vs 26.6 ± 8.76 mm^2^, respectively. However, after 96 h, *flg*-Δ3 OFF strains exhibited significantly higher motility than *fliC* strains (*P* = 0.0043, Mann-Whitney test) with areas of motility of 204 ± 116 mm^2^ vs 35.6 ± 11.6 mm^2^, respectively (Fig. 3C). Overall, the results support a binary model of swimming motility over the first 48 h with the wild-type, *pilA1*, and *flg*-Δ3 ON strains showing similar motility, while the *fliC, fliD*, and *flg*-Δ3 OFF mutants were essentially non-motile.

### Flagellar mutants are defective in adhesion to mucosal hydrogels

**Fig 4:**
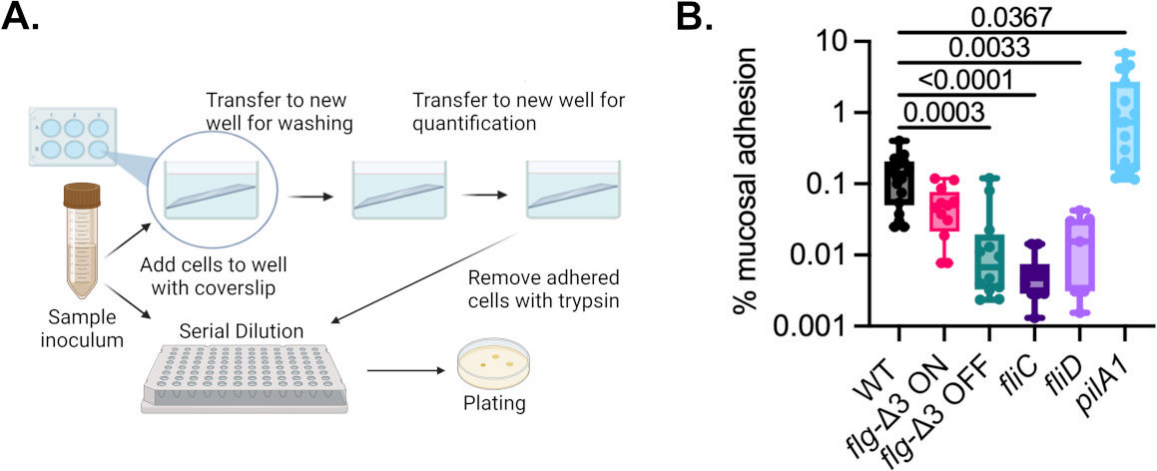
*C. difficile* binds specifically to mucin hydrogels *in vitro*. (**A**) Overview of approach to quantify mucin-specific binding through selective plating. (**B**) Comparison of wild-type and mutant *C. difficile* R20291 adhered to mucin hydrogels. Significance of differences from wild type was determined by Brown-Forsythe and Welch ANOVA test with Dunnett’s T3 correction for multiple comparisons.

Previously, we observed that the *fliC* mutant exhibited impaired binding to mucosal hydrogels formed on glass surfaces, while the *pilA1* mutant showed a modest increase in binding (22). Given that *fliC* is not only a structural protein of flagella, but also part of a regulatory network with levels potentially upregulating surface adhesins, we evaluated mucosal adhesion in other mutant strains (*fliD, flg*-Δ3 ON*,* and *flg*–Δ3 OFF) with altered levels of flagellation to determine whether variation in other flagellar components might impact mucosal binding (37). For wild-type R20291 cells, we observed that 0.14 ± 0.099% of cells bound to mucin after a 1 h incubation (Fig. 4). In line with our previous results, *pilA1* showed increased binding at 1.5± 2.0%, whereas the *fliC* mutant showed essentially no binding (0.0060 ± 0.0046%). Similarly, *fliD* (0.018 ± 0.017%) and *flg*-Δ3 OFF (0.023 ± 0.037%) demonstrated reduced binding compared to the wild type. However, in contrast to the *pilA1* mutant, *flg*-Δ3 ON (0.052 ±0.037 %) showed a slight reduction in binding relative to the parent strain rather than increased binding as hypothesized.

### Loss of flagellation decreases adherence to mucin-secreting cells in 2D culture

**Fig 5:**
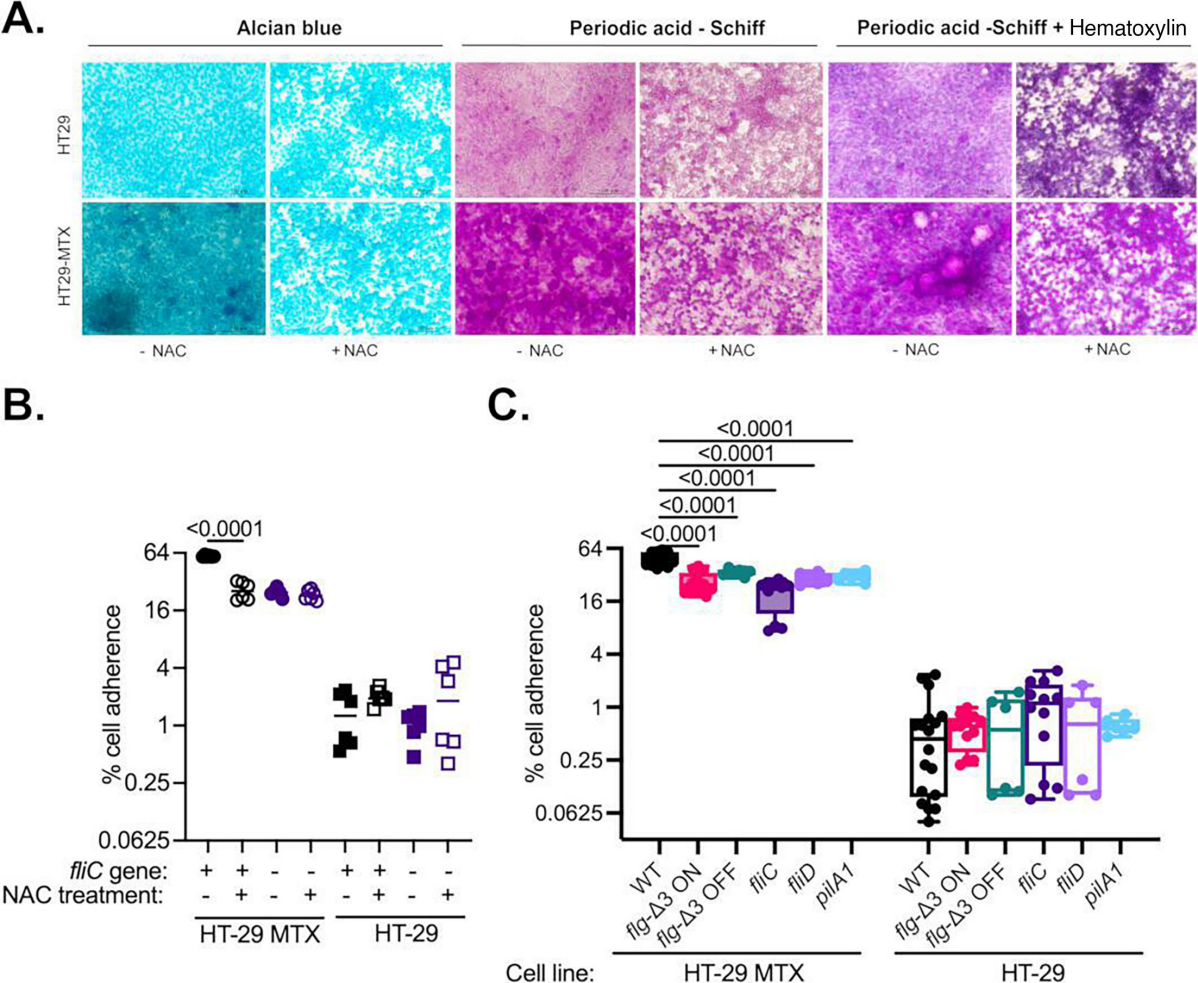
Adherence to HT-29 and mucin-producing HT-29 MTX cells. (**A**) HT-29 and HT-29 MTX cells stained with Alcian Blue, which stains acidic mucins, periodic acid-Schiff (PAS), which stains neutral mucins, and PAS with hematoxylin, which stains nuclei, with and without treatment with the mucolytic agent NAC. (**B**) Percentage adherence of wild-type (+) and *fliC* (-) strains to HT-29 and HT-29 MTX cells that were untreated or treated with the mucolytic agent NAC. Significance of differences from wild-type cells adhered to NAC-treated cells (HT-29 MTX or HT-29) was determined by one-way ANOVA with Brown-Forsythe and Welch’s correction for unequal variances and Dunnett’s T3 correction for multiple comparisons. (**C**) Percentage adherence of indicated *C. difficile* strains to HT-29 or HT-29 MTX cells. Significance of differences between wild-type and mutant strain binding for each cell line (HT-29 or HT-29 MTX) was determined by one-way ANOVA with Brown-Forsythe and Welch’s correction for unequal variances and Dunnett’s T3 correction for multiple comparisons.

Previous studies measuring adherence by *C. difficile* to 2D Caco-2 cell monolayers showed strain-dependent defects in binding for *fliC* mutants; a defect was found in the R20291 background (22), but not in CD630 (30). Because cells in 2D culture can both secrete mucins and/or express membrane-bound mucins on their surfaces, this defect could also be explained as a defect in mucosal adhesion. To test this hypothesis, we compared the adhesion of *C. difficile* R20291 wild-type and *fliC* bacteria to monolayers of HT-29 and HT-29 MTX cells. The HT-29 cell line was derived from a colorectal adenocarcinoma (38), and HT-29 MTX cells were derived as a stable subpopulation from HT-29 cells after treatment with methotrexate (39). This model has been used previously to measure the impact of mucus secretion on adherence by anaerobic bacteria (40). We also previously found that the R20291 *fliC* mutant has a defect in adhesion to mucosal hydrogels formed with mucins purified from HT-29 MTX cells compared to the parent strain (22). Consistent with previous observations, we found that HT-29 MTX cells formed thicker mucus layers than HT-29 cells as measured by mucin staining (Fig. 5, panel A) and that adherence of R20291 WT cells to HT-29 MTX cells (67 ± 5.5%) was >180-fold higher than adherence to HT-29 cells (0.37 ± 0.038%) (Fig. 5). Binding of the *fliC* mutant to HT-29 MTX cells (32 ± 2.0%) was significantly lower than WT cells, whereas there were no significant differences in binding to HT-29 cells (0.53 ±0.052%) between WT and *fliC* mutants. Treatment with N-acetyl cysteine (NAC), to decrease levels of mucus on the cell surface, reduced the adherence of WT to the level of the *fliC* mutant and had no further effect on adhesion by the *fliC* mutant. However, adhesion by both WT and *fliC* was higher to HT-29 MTX cells than HT-29 cells even after NAC treatment (Fig. 5, panel B). In a direct comparison of *C. difficile* R20291 WT to flagellar and T4P mutants, all mutant strains (*flg*-Δ3 ON, *flg*-Δ3 OFF, *fliC*, *fliD,* and *pilA1*) showed decreased adherence to HT-29 MTX cells but equivalent, lower, adherence to HT-29 cells. In this model, centrifugation of *C. difficile* onto cell monolayers decreased adherence for both wild-type and *fliC* (Fig. S2). These results provide further support for the importance of the mucin layer as a site for *C. difficile* attachment and demonstrate that *fliC* contributes to this adhesion, but also highlight that adhesion to cell monolayers is more complex than adhesion to mucus alone.

### Hyperflagellation is not mediated by increased expression of *fliC*

**Fig 6:**
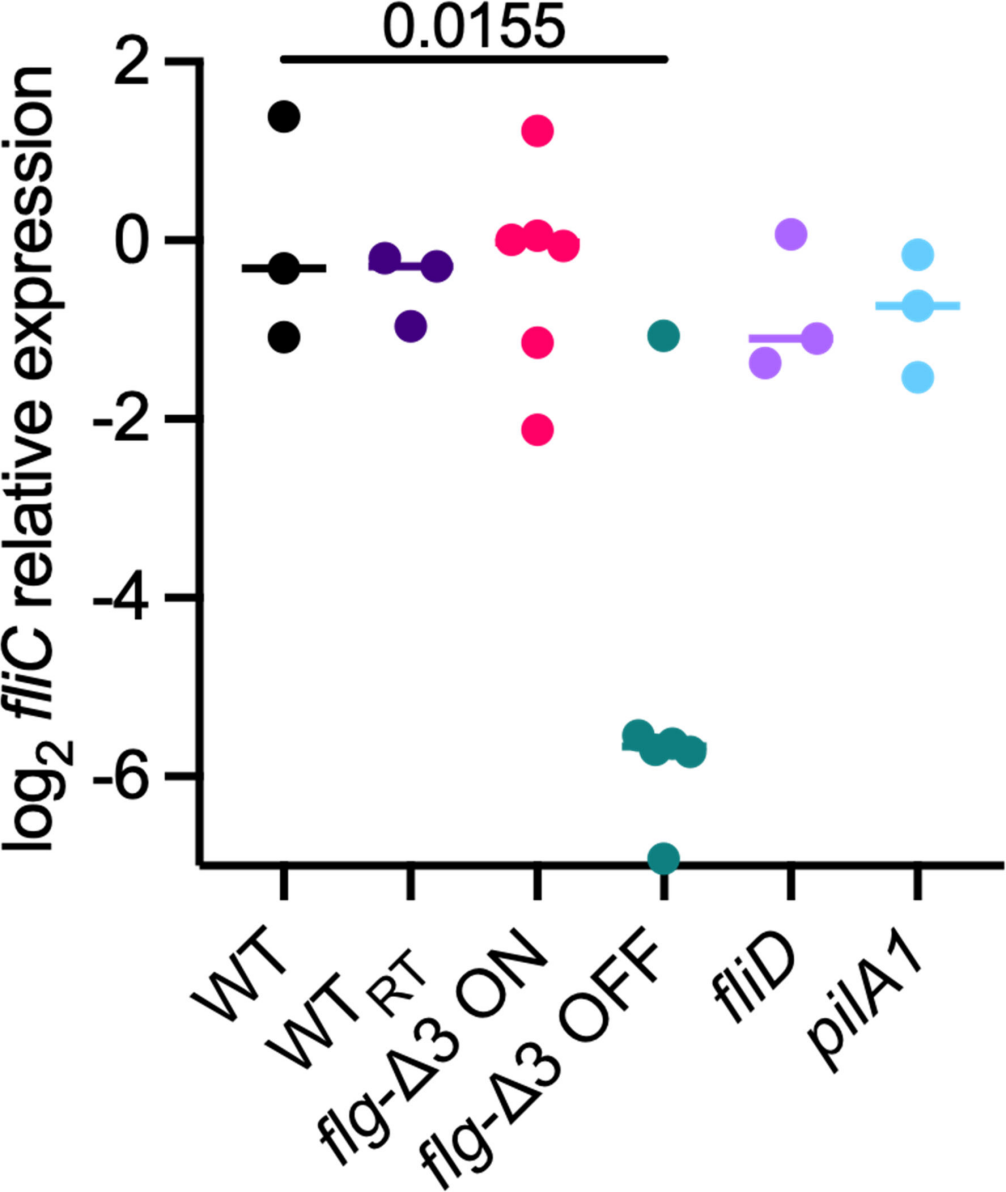
Impacts of mutations on *fliC* gene expression. Comparisons of relative levels of *fliC* gene expression between R20291 wild type (received from either Glen Armstrong’s group [WT] or Rita Tamayo’s group [WT_RT_]), *pilA1, fliC, fliD* (all derived from WT)*,* and *flg*-Δ3 ON and *flg*-Δ3 OFF (derived from WT_RT_) through qRT-PCR. Statistical significance from wild type was determined by one-way ANOVA with Brown-Forsythe and Welch correction for unequal variances and Dunnett’s T3 correction for multiple comparisons.

One potential mechanism for hyperflagellation in *pilA1* and *flg*-Δ3 ON mutants could be increased expression of *fliC*. We used qRT-PCR to compare *fliC* transcription levels across *C. difficile* strains. We grew R20291 wild-type and the *pilA1*, *flg*-Δ3 ON, flg-Δ3 OFF, and *fliD* mutant strains and measured *fliC* expression by qRT-PCR, normalizing the results to the average expression level in the wild-type strain. Transcription of *fliC* varied across replicate samples collected from wild-type strains, with a mean level of log_2_ normalized expression of 0 ± 1.3 (Fig. 6). No significant differences in *fliC* expression were observed between *fliD* (−0.8 ± 0.8), *flg*-Δ3 ON (−0.3 ± 1.2), or *pilA1* (−0.8 ± 0.7) and the parent strain. However, *flg*-Δ3 OFF (−5.0 ± 2.0) exhibited significantly lower *fliC* expression compared to wild-type. These trends were not impacted by time in broth culture; equivalent results were obtained from cells directly inoculated from agar plates and from cells grown in broth culture overnight before inoculation (Fig. S3). Because *flg*-Δ3 ON and flg-Δ3 OFF were obtained from the Tamayo lab and are of the same lineage but may have experienced genetic drift relative to our strains, we also compared *fliC* expression in our R20291 wild-type strain to the R20291 wild-type strain obtained directly from Rita Tamayo’s lab, wild-type_RT_. However, no significant differences in *fliC* expression were observed between the two wild-type strains.

## DISCUSSION

The high rates of recurrence, the emergence of hypervirulent strains, and the ongoing increase in the incidence of *C. difficile* underscore the necessity of identifying pharmacological targets and developing innovative therapeutic approaches to treat *C. difficile* (41). However, the identification of specific molecular mechanisms critical for *C. difficile* colonization is complicated by the interplay between host-pathogen and inter-microbial interactions (41). The gut microbiota disruption created by antibiotics creates conditions favorable for *C. difficile* colonization and toxin production (42–49). Although it is known that *C. difficile* exploits an intestinal environment after disruption of the commensal microbiota, the precise mechanism of these host-microbe and inter-microbial interactions is unknown (50). Multiple studies have shown that *C. difficile* adheres to host-derived cells in 2D culture (51–58); more recently, strong interactions have been observed in *C. difficile* adherence to mucus both *in vivo* (23) and *ex vivo* (22). Competition between microbes for host mucins as attachment sites and microbial degradation of host mucins as nutrient sources are potential factors influencing the competition between *C. difficile* and the commensal microbiome. These interactions with host mucosa could provide another avenue for commensal microbes to contribute to *C. difficile* colonization.

Previous studies of *C. difficile* R20291 have shown that flagellin is a key component of *C. difficile* adherence and persistence (21, 59), although similar experiments in CD630 have not shown equivalent effects (30). In addition to serving as the major structural component of flagella, FliC also contributes to the complex regulatory network that governs activities related to colonization, virulence, and toxin gene expression (21, 37, 60–62). Expression of *fliC,* toxin synthesis, bacterial movement, colonization, and pathogenicity are intricately regulated through a network that includes the global regulator CsrA (carbon storage regulator protein A). Previous studies have shown that FliW influences *fliC* expression by obstructing CsrA-mediated post-transcriptional regulation (24). Additionally, FliW has been shown to suppress CsrA *in vivo*, which may have detrimental effects on *C. difficile* pathogenicity (24). Additional factors that regulate FliW, CsrA, and FliC, as well as the function of FliW in *C. difficile,* are still unknown, even though the critical functions of CsrA in flagellin synthesis and flagellin homeostasis have been investigated in other bacteria (63). Furthermore, flagellum and toxin expression are linked through the flagellar alternative sigma factor, SigD, which is encoded in the F3 operon (64).

Phase variation of the F3 operon also contributes to the regulation of flagella and toxin expression (25, 65). However, the role that this regulation plays in pathogenesis is complex. While phase-locked *flg*-Δ3 ON mutants accumulated more toxins than the phase-locked *flg*-Δ3 OFF mutants in a hamster model of acute *C. difficile* infection, they were not substantially different in their capacity to induce acute illness symptoms. In mice, *flg*-Δ3 ON mutants showed both greater persistence and greater morbidity (25), but the *flg*-Δ3 OFF mutant colonized at levels intermediate between wild-type and *flg*-Δ3 ON. *flg*-Δ3 ON mutants have also previously been shown to be flagellated and motile, unlike their *flg*-Δ3 OFF counterparts (26).

Recently, we demonstrated that both O-linked glycosylation of host mucins and *C. difficile fliC* were required for mucin adherence by *C. difficile (22*). While we hypothesized that direct interactions between flagella and mucins were responsible for this adherence mechanism, an alternative hypothesis is that the synthesis of downstream lectin-like adhesins is controlled by the expression of *C. difficile* FliC. Here, we have demonstrated that mutants lacking flagella but not *fliC* (*fliD*, *flg*-Δ3 OFF) also exhibited reduced mucin binding. Conversely, while both *pilA1* and *flg*-Δ3 ON mutants were hyperflagellated, we observed that only *pilA1* mutants exhibited increased mucin binding. These results indicate that while flagellation is one factor contributing to mucin adherence, other factors likely contribute to mucin adherence in the *pilA1* mutant. Similarly, despite being hyperflagellated, *pilA1* and *flg*-Δ3 ON mutants show similar *fliC* expression levels to the parent strain, indicating that hyperflagellation is not regulated through transcription of *fliC*. Furthermore, the *flg*-Δ3 OFF mutant strain is the only strain we tested to show lower levels of *fliC* expression compared with the parent strain; the *fliD* mutant is aflagellate but shows similar levels of *fliC* transcription to the parent strain. This result is similar to what was previously observed for naturally occurring R20291 phase variants (26) but differs from a prior comparison of phase variants in CD630, which showed both flagellate and aflagellate derivatives had lower *fliC* transcription than the parent strain (65). These results suggest that *fliC* transcription may be differentially regulated between CD630 and R20291, and that through the F3 operon, expression of other flagellar components is necessary for robust *fliC* transcription. The origin of the hyperflagellation seen in the *flg*-Δ3 ON and *pilA1* mutants is not clear but could stem from regulation of other flagellar genes (including those in the F3 operon) or post-transcriptional control of FliC levels.

Our observation that *fliC* contributes to mucin adherence was not limited to binding to synthetic mucosal hydrogels, as we also observed defects in *fliC* adherence to HT-29 MTX cells, a 2D cell culture model that was previously shown to support mucin-specific adherence of *Clostridium perfringens (40*). In contrast, there were no significant differences in binding between WT and *fliC* cells to HT-29 MTX cells treated with a mucolytic agent or to HT-29 cells that produce low levels of secreted mucins. A previous report has also shown an approximately fivefold reduction in binding of an *fliC* strain to Caco-2 monolayers compared to wild-type (61). Caco-2 cells produce MUC1, MUC3, MUC4, and MUC5A/C (66), whereas HT-29 MTX cells produce high levels of MUC5A/C and low levels of MUC2 (31). Differences in the effects of *fliC* mutants in these assays could possibly be due to the types of mucins produced or variations between *C. difficile* strains. Further studies are needed to explore these differences more fully. In particular, we note that the difference in phenotype for the *pilA1* mutant, which shows increased adhesion to mucosal hydrogels but decreased adhesion to HT-29 MTX cells, implies that multiple modes of adhesion exist for the attachment of *C. difficile* to host tissues. Previously, McKee found no significant difference in adhesion to HT-29 or MDCK cells for a CD630 *pilA1* mutant after 1 h, but a decrease in adhesion to MDCK cells after 24 h (67). Taken together, these results imply that multiple processes are likely to be important for understanding *C. difficile* host attachment in a physiological context, which involves multiple cell types, secreted and membrane-bound mucins, and variable time scales.

Based on these combined results, we conclude that flagellation is not generally limited or controlled by levels of *fliC* transcription but by other factors (including the expression of other flagellar genes in the F3 operon) and that flagellar adhesion alone cannot explain mucosal adherence by *C. difficile*. Instead, these findings suggest that *C. difficile* mucosal adherence is influenced by multiple molecular mechanisms (8), some of which are dependent upon flagellation, but also include unknown factors that differ between naturally occurring strains. Here, these differences are highlighted in the differences between the hyperflagellated *flg*-Δ3 ON and *pilA1* mutants. A comprehensive model of *C. difficile* host colonization will require identification of both the intermolecular contacts between *C. difficile* and its hosts, as well as the variation in *C. difficile* physiology throughout the course of infection.

## Data Availability

Genomes of R20291 strains from Glenn Songer and Rita Tamayo, as well as two variants of putative *flg*-Δ3 ON, were sequenced with short-read, whole-genome Illumina sequencing performed by SeqCenter LLC (Pittsburgh, PA). Data are available at NCBI SRA under accession numbers SAMN51298324, SAMN51298325, SAMN51298326, and SAMN51298327.

## SUPPLEMENTAL MATERIAL

Supplemental figures jb.00428-25-s0001.docx Figures S1 to S3.

ASM does not own the copyrights to Supplemental Material that may be linked to, or accessed through, an article. The authors have granted ASM a non-exclusive, world-wide license to publish the Supplemental Material files. Please contact the corresponding author directly for reuse.
