# Supplementary material for: Dissecting the role of flagellar subunits in C. difficile mucosal colonization
Source: J Bacteriol. 2025 Nov 24;207(12):e00428-25. doi: 10.1128/jb.00428-25 (PMC12713411; doi:10.1128/jb.00428-25)
Supplement: Supplemental figures — Figures S1 to S3. [file jb.00428-25-s0001.docx]

**Supporting Figure 1: Flagellation of *C. difficile* from liquid and solid media.** A) Average number of flagella per cell, B) proportion of flagellated cells. *C. difficile* in broth culture were grown, fixed and analyzed by TEM as described in Methods. Bacteria grown on BHIS (without cysteine) plates were scrapped and gently resuspended in fixative solution before being applied to TEM grids.


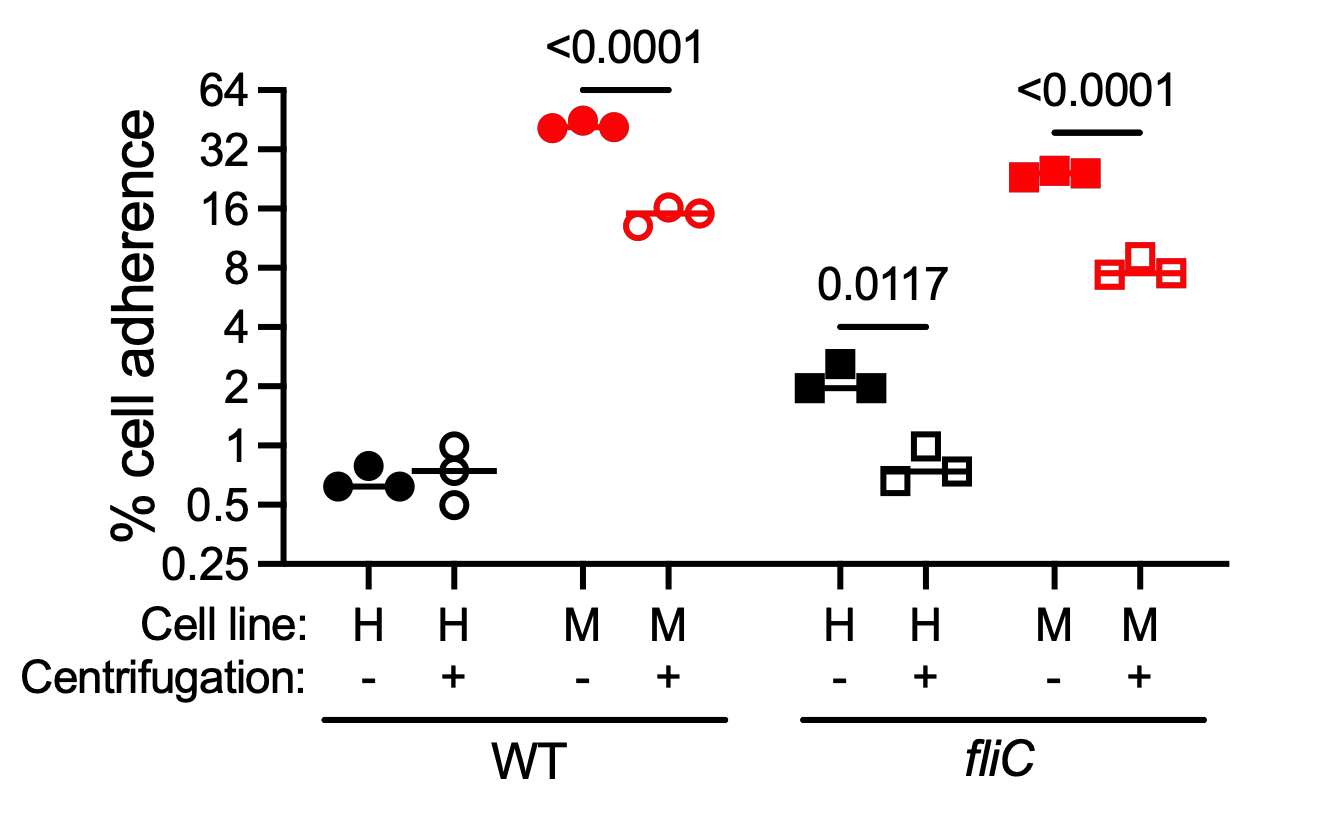


**Supporting Figure 2:** **Centrifugation decreases *C. difficile* adherence.** Adherence of wild-type and *fliC* *C. difficile* strains to HT29 and HT29-MTX cells were measured with or without centrifugation to promote settling of non-motile *fliC* cells. Significance was determined by student’s t-test with Welch’s correction for unequal variance.


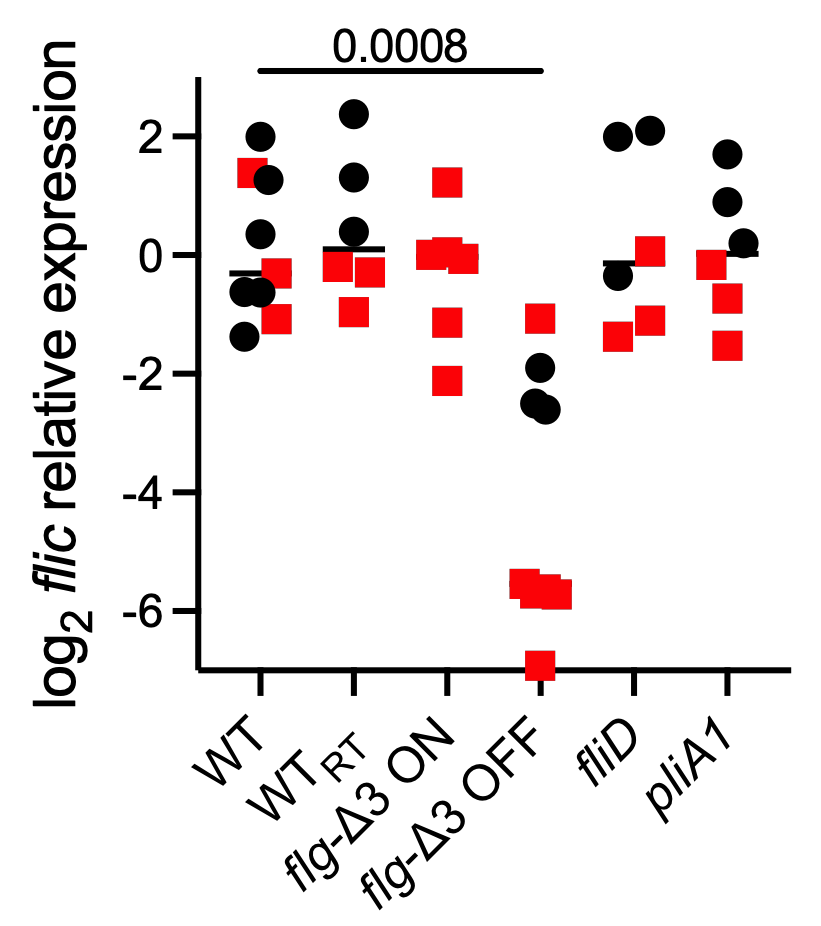


**Supporting Figure 3:** **Decreased *fliC* relative expression in *flg-3* OFF strains not impacted by time in broth culture.** *fliC* relative expression levels were determined through qRT-PCR for cells that were grown overnight in broth before growth in fresh medium for four generations (red squares; data shown in Figure 6) or for cells inoculated directly into fresh broth from petri plates and grown for approximately four generations (black circles). Statistical significance from wild-type was determined by one-way ANOVA with Brown-Forsythe and Welch correction for unequal variances and Dunnett T3 correction for multiple comparisons.
